# Frequency Characteristics of Pulse Wave Sensor Using MEMS Piezoresistive Cantilever Element

**DOI:** 10.3390/mi13050645

**Published:** 2022-04-19

**Authors:** Taiga Nabeshima, Thanh-Vinh Nguyen, Hidetoshi Takahashi

**Affiliations:** 1 Department of Mechanical Engineering, Faculty of Science and Technology, Keio University, 3-14-1 Hiyoshi, Kouhoku-ku, Yokohama 223-8522, Kanagawa, Japan; ti9er@keio.jp; 2 Sensing System Research Center, National Institute of Advanced Industrial Science and Technology (AIST), 1-2-1 Namiki, Tsukuba 305-8564, Ibaraki, Japan; vinh.nguyen@aist.go.jp

**Keywords:** pulse wave sensor, piezoresistive cantilever, frequency characteristics

## Abstract

Wearable sensor devices with minimal discomfort to the wearer have been widely developed to realize continuous measurements of vital signs (body temperature, blood pressure, respiration rate, and pulse wave) in many applications across various fields, such as healthcare and sports. Among them, microelectromechanical systems (MEMS)-based differential pressure sensors have garnered attention as a tool for measuring pulse waves with weak skin tightening. Using a MEMS-based piezoresistive cantilever with an air chamber as the pressure change sensor enables highly sensitive pulse-wave measurements to be achieved. Furthermore, the initial static pressure when attaching the sensor to the skin is physically excluded because of air leakage around the cantilever, which serves as a high-pass filter. However, if the frequency characteristics of this mechanical high-pass filter are not appropriately designed, then the essential information of the pulse-wave measurement may not be reflected. In this study, the frequency characteristics of a sensor structure is derived theoretically based on the air leakage rate and chamber size. Subsequently, a pulse wave sensor with a MEMS piezoresistive cantilever element, two air chambers, and a skin-contacted membrane is designed and fabricated. The developed sensor is 30 mm in diameter and 8 mm in thickness and realizes high-pass filter characteristics of 0.7 Hz. Finally, pulse wave measurement at the neck of a participant is demonstrated using the developed sensor. It is confirmed that the measured pulse wave contains signals in the designed frequency band.

## 1. Introduction

Pulse wave is one of the four basic human vital signs, and it indicates the state of a human body’s life-support functions [1,2,3,4,5]. Conventionally, the pulse wave is measured while the patient is positioned in front of an unportable measurement equipment. However, parallel with advances in technology, wearable sensor devices with minimal discomfort to the wearer have been widely developed to realize continuous pulse wave measurements in many applications across various fields, such as healthcare and sports [6,7,8,9,10]. The most advanced wearable sensor devices for pulse-wave measurement are optical and mechanical sensors. Optical sensors measure pulse waves by detecting light transmission and reflection on the skin surface above the blood vessel via light-emitting diodes and photodiodes [11,12]. Even though the effects of skin tone and pigmentation are non-negligible [13,14], optical sensors are suitable for miniaturization because of their simple components; hence, they have been used in commercial smartwatches and pulse oximeters. By contrast, mechanical sensors measure pulse waves by detecting pulse-induced skin deformation above the blood vessel, which typically involves strain or pressure [15,16,17,18,19,20,21,22,23,24,25,26]. Mechanical detection provides direct information regarding physical phenomena occurring in the blood vessels. Strain sensors are typically fabricated using flexible polymers and can be attached to the skin directly [15,16,17,18]. Meanwhile, pressure sensors utilize silicon-based sensor components with high functionalities. These sensors measure pulse-induced skin deformation through an elastic body [19,20,21], liquid [22], or air chamber [23,24,25,26] with minimal contact force. The solid sensor component offers practicability owing to its high stability and ease of mass production. The skin deformation varies with age and other individual differences, but the absolute values are not so meaningful for pulse wave measurements.

Among pressure sensor types, the microelectromechanical systems (MEMS) cantilever-type differential pressure sensor with an air chamber can achieve a much high sensitivity; moreover, when it is attached to the skin, its initial static pressure is physically excluded because of the air leakage around the cantilever, which serves as a mechanical high-pass filter [24,26]. When measuring the pulse wave, the frequency characteristics of the sensor must be designed such that the sensor can detect important information, such as minor peaks in the pulse wave. Therefore, the process by which the frequency characteristics of the sensor are determined using structural parameters must be clarified to achieve an appropriate design.

**Figure 1:**
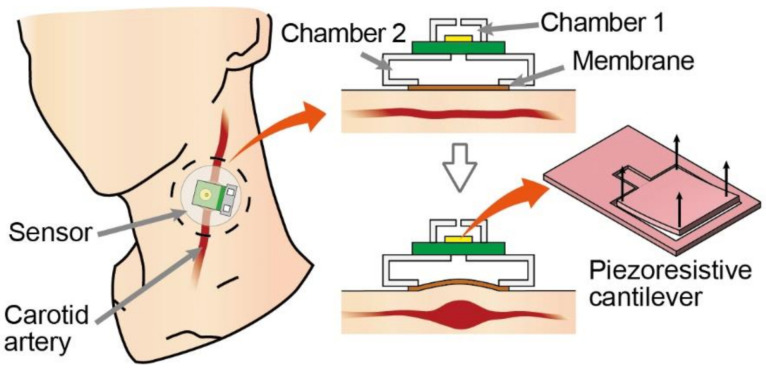
Schematic illustration of pulse wave sensor. Pulse wave sensor is composed of MEMS piezoresistive cantilever and two air chambers. One of the two chambers is pressed against the skin via a membrane to detect vibrations caused by pulse waves.

Herein, we propose a pulse wave sensor that uses MEMS cantilever-type differential pressure sensor as the sensing element and exhibits the appropriate frequency characteristics. Specifically, the proposed sensor is composed of a piezoresistive cantilever, two air chambers, and a skin-attached membrane, as shown in Figure 1. The membrane deforms owing to skin deformation caused by pulse waves, resulting in a change in the one-chamber volume. Because the chamber pressure varies with volume change, the pulse wave can be measured by detecting the differential pressure between the two chambers. The sensor exhibits high-pass filter characteristics that are the same as those reported previously [24]. The frequency response of the high-pass filter depends on the air leakage rate and volume of the two chambers. First, we theoretically derived the frequency characteristics based on the abovementioned parameters. Subsequently, we designed and fabricated a pulse wave sensor suitable for the pulse wave frequency response. Finally, we demonstrated pulse wave measurements by attaching the developed sensor to the neck of a participant.

## 2. Design and Principle

### 2.1. Sensor Design

The pulse waves of humans comprise signals of multiple frequencies, with the lowest frequencies in the range of 0.8–1.7 Hz [27]. Hence, the cutoff frequency of the high-pass filter of the sensor should be less than 0.8 Hz. Additionally, the measurement point is assumed to be at the carotid artery, which is the typical location for measuring pulse waves, and the size of the sensor should be minimal to avoid discomfort when attached to the neck. In this study, we designed the sensor to be less than 40 mm in diameter to prioritize ease of fabrication.

**Figure 2:**
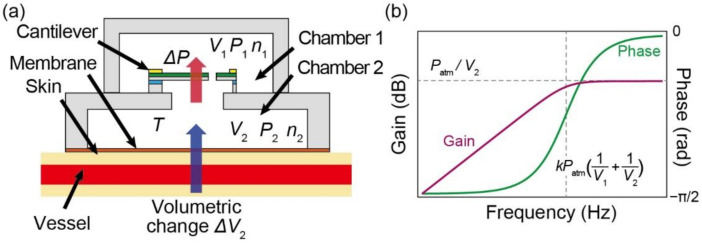
(**a**) Design parameters and (**b**) theoretical frequency characteristics of proposed sensor.

The details of the sensor design are shown in Figure 2a. The sensor is composed of a MEMS piezoresistive cantilever and two air chambers: Chamber 1 and Chamber 2. Chamber 2 was covered with a 50 μm-thick polyimide membrane that was in contact with the skin during the measurement. The cantilever was placed between Chambers 1 and 2 to measure the differential pressure between them. Because the membrane was soft, small deformations of the skin surface caused by pulse waves were directly transformed into deformations on the membrane. Consequently, a pressure change occurred in Chamber 2. The pulse wave can be measured by detecting the difference between the pressures of the two chambers. Because of air leakage through the gap surrounding the cantilever, the cantilever response resembled that of a mechanical high-pass filter, which allows the physical elimination of static pressure caused by the initial deformation of the membrane when the sensor is pressed against the skin. The characteristics of the high-pass filter are governed by the volume of the chambers. We denote the pressure, volume, and amount of substance (air) of Chambers 1 and 2 as *P*_1_, *V*_1_, *n*_1_, *P*_2_, *V*_2_, and *n*_2_, respectively, as shown in Figure 2a. In addition, the volumetric change owing to the pulse wave is denoted as Δ*V*_2_.

### 2.2. Theoretical Frequency Characteristics

The sensor frequency characteristics were theoretically derived. First, the Boyle–Charles equation of state holds in the two chambers. The differential pressure Δ*P* is defined as the pressure difference between the two chambers as follows:

(1) $$\left\{\begin{array}{l} P_{1}V_{1}=n_{1}RT\\ P_{2}V_{2}=n_{2}RT\\ \Delta P=P_{2}-P_{1,} \end{array}\right.$$

where *R* and *T* are the gas constant and temperature inside the chamber, respectively. The volume flow rate *q*, which is reciprocated by air leakage around the cantilever, is expressed as follows:

(2) $$\left\{\begin{array}{l} \frac{dn_{1}}{dt}=q\frac{n_{1}}{V_{1}}\\ \frac{dn_{2}}{dt}=-q\frac{n_{2}}{V_{2}} \end{array}\right.$$

Here, we assume that the volume flow rate *q* is proportional to Δ*P*, provided that the cantilever gap is sufficiently narrow such that the air leakage rate is low. Therefore, it can be expressed as follows using the proportionality coefficient, *k*:

(3) q=kΔP

The proportionality coefficient *k*, an indicator of air leakage, is a parameter that varies with the cantilever dimensions. The following equation was derived using Equations (2) and (3):

(4) $$\left\{\begin{array}{l} \frac{dn_{1}}{dt}=k\frac{n_{1}}{V_{1}}\Delta P\\ \frac{dn_{2}}{dt}=-k\frac{n_{2}}{V_{2}}\Delta P \end{array}\right.$$

By differentiating the upper equation in Equation (1) with respect to time *t* and then substituting Equation (4) into the result, the relationship between the pressure change *dP*_1_/*dt* in Chamber 1 and Δ*P* is obtained as follows:

(5) $$\begin{array}{c} \frac{dP_{1}}{dt}=\frac{dn_{1}}{dt}\frac{RT}{V_{1}}\\ =k\frac{P_{1}}{V_{1}}\Delta P \end{array}$$

Based on Equation (1), because *P*_2_ = *P*_1_ + ∆*P*, the following equation is obtained:

(6) $$\frac{dP_{2}}{dt}=\left(k\frac{P_{1}}{V_{1}}+\frac{d}{dt}\right)\Delta P$$

Similarly, the middle equation in Equation (1) can be transformed as follows:

(7) $$\left\{\begin{array}{c} P_{2}\frac{dV_{2}}{dt}+V_{2}\frac{dP_{2}}{dt}=\frac{dn_{2}}{dt}RT=-k\Delta P\cdot \frac{n_{2}RT}{V_{2}}\\ \frac{dP_{2}}{dt}=-k\frac{P_{2}}{V_{2}}\Delta P-\frac{P_{2}}{V_{2}}\frac{dV_{2}}{dt} \end{array}\right.$$

Subsequently, substituting Equation (6) into Equation (7) yields

(8) $$\left\{\begin{array}{c} -k\frac{P_{2}}{V_{2}}\Delta P-\frac{P_{2}}{V_{2}}\frac{dV_{2}}{dt}=\left(k\frac{P_{1}}{V_{1}}-\frac{d}{dt}\right)\Delta P\\ \frac{dV_{2}}{dt}=\frac{\left(\frac{d}{dt}-k\frac{P_{1}}{V_{1}}-k\frac{P_{2}}{V_{2}}\right)}{\frac{P_{2}}{V_{2}}}\Delta P \end{array}\right.$$

From the viewpoint of a single input/output system, Δ*P* and the lower chamber volume change Δ*V*_2_ correspond to the output and input, respectively. Therefore, Equation (8) can be expressed as the following simple transfer function *G*_1_(*s*) using the Laplace operator *s*:

(9) $$G_{1}\left(s\right)=\frac{\Delta P}{\Delta V_{2}}=\frac{\frac{P_{2}}{V_{2}}s}{s-k\frac{P_{1}}{V_{1}}-k\frac{P_{2}}{V_{2}}}=\frac{P_{\mathrm{atm}}}{V_{2}}\cdot \frac{s}{s-kP_{\mathrm{atm}}\left(\frac{1}{V_{1}}+\frac{1}{V_{2}}\right)}$$

where it is assumed that Δ*P* is small, and *P*_1_ and *P*_2_ are of the same order as the atmospheric pressure *P*_atm_. Based on Equation (9), the gain and phase characteristics of the Bode plot of the input/output system can be described, as shown in Figure 2b. These frequency characteristics are those of a high-pass filter with a cutoff frequency *f*, expressed as

(10) $$f=kP_{\mathrm{atm}}\left(\frac{1}{V_{1}}+\frac{1}{V_{2}}\right)$$

Therefore, the values of *V*_1_ and *V*_2_ determine the gain characteristics and cutoff frequency of the sensor.

To obtain accurate pulse wave measurements, all frequency components in the pulse wave should be measured. Equation (9) suggests that both *V*_1_ and *V*_2_ should be maximized to reduce the cutoff frequency such that all the frequency components of the pulse wave are in the pass band of the high-pass filter. By contrast, the sensor sensitivity is inversely proportional to *V*_2_ as the inverse of *V*_2_ is on the right side of Equation (9). In addition, the sensor should be as compact as possible, considering wearables. Based on this analysis, we discovered that the optimal solution is to render the volume of the upper chamber *V*_1_ effectively infinite by connecting it to the outside air. Consequently, the effect of the upper chamber can be disregarded, and Equations (9) and (10) become:

(11) $$\left\{\begin{array}{c} G_{1}\left(s\right)=\frac{P_{\mathrm{atm}}}{V_{2}}\cdot \frac{s}{s-\frac{kP_{\mathrm{atm}}}{V_{2}}}\\ f=\frac{kP_{\mathrm{atm}}}{V_{2}} \end{array}\right.$$

Based on Equation (11), the cutoff frequency varies depending only on *V*_2_. As mentioned in Section 2.1, the lowest frequency component in the pulse wave is approximately 0.8 Hz, and the cutoff frequency of the sensor high-pass filter should be lower than this value. Hence, we set the cutoff frequency of the high-pass filter to 0.7 Hz, which is lower than the lowest frequency component, and *V*_2_ can be expressed as follows:

(12) $$V_{2}=\frac{kP_{\mathrm{atm}}}{0.7}$$

If the constant *k* is known, then the optimum lower chamber volume *V*_2_ can be obtained to design a pulse wave sensor using a cantilever.

### 2.3. Frequency Calibration Method

**Figure 3:**
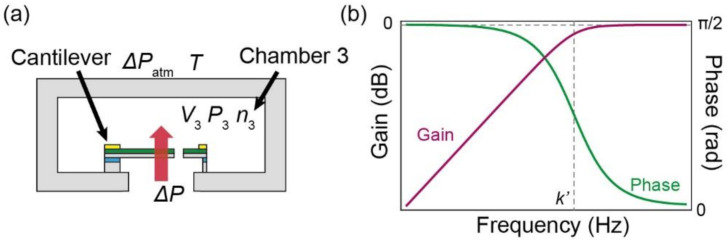
(**a**) Calibration method to evaluate frequency response of piezoresistive cantilever. (**b**) Theoretical frequency characteristics based on calibration.

To design the frequency characteristics of the sensor, the constant *k* associated with the air leakage of the cantilever must be determined. This constant is calculated theoretically using the abovementioned approach based on the experimental configuration, which comprises a cantilever and a single air chamber, as shown in Figure 3a. It is derived when the external pressure change is the input and the cantilever response against the differential pressure is the output. In this configuration, similar to Equations (1) and (5), the Boyle–Charles equation of state holds in the chamber, as follows:

(13) $$\left\{\begin{array}{c} P_{3}V_{3}=n_{3}RT\\ \frac{dP_{3}}{dt}=\frac{dn_{3}}{dt}\frac{RT}{V_{3}}, \end{array}\right.$$

where *P*_3_, *V*_3_, and *n*_3_ denote the pressure, volume, and amount of substance inside the chamber, respectively. Additionally, the pressure changes are defined as follows:

(14) $$\left\{\begin{array}{c} P_{\mathrm{atm}}=P_{0}+\Delta P_{\mathrm{atm}}\\ P_{3}=P_{0}+\Delta P_{3}\\ \Delta P^{\prime }=P_{\mathrm{atm}}-P_{3} \end{array}\right.$$

At this time, the volume flow rate *q* satisfies the following equation:

(15) $$\frac{dn_{3}}{dt}=q\frac{n_{3}}{V_{3}}$$

By substituting Equation (15) and the proportionality constant *k* into the lower equation in Equation (13), the following relationship is obtained:

(16) $$\frac{d\Delta P_{3}}{dt}=k\frac{P_{0}}{V_{3}}\Delta P\;$$

This equation can be transformed to the following:

(17) $$\left\{\begin{array}{c} \frac{d}{dt}\Delta P_{\mathrm{atm}}=\left(\frac{d}{dt}+k\frac{P_{0}}{V_{3}}\right)\Delta P\\ \frac{\Delta P}{\Delta P_{\mathrm{atm}}}=\frac{\frac{d}{dt}}{\frac{d}{dt}+k\frac{P_{0}}{V_{3}}} \end{array}\right.$$

Provided that this system is an input–output system with the atmospheric pressure change Δ*P*_atm_ as the input and the differential pressure Δ*P* as the output, Equation (17) can be expressed by the following transfer function *G*_2_(*s*) using the Laplace operator *s*:

(18) $$G_{2}\left(s\right)=\frac{s}{s+k^{\prime }}\;\;\;\;\;\;\;\;\;\;\left(k^{\prime }=k\frac{P_{0}}{V_{3}}\right)$$

Based on Equation (18), the gain and phase characteristics of the Bode plot of the input–output system can be expressed, as shown in Figure 3b. The frequency characteristics are those of a simple high-pass filter with a cutoff frequency of *k*′. By experimentally obtaining the Bode plot using a piezoresistive cantilever and a chamber of volume *V*_3_, the constant *k* can be calculated using the cutoff frequency *k*′ obtained.

### 2.4. Piezoresistive Cantilever

**Figure 4:**
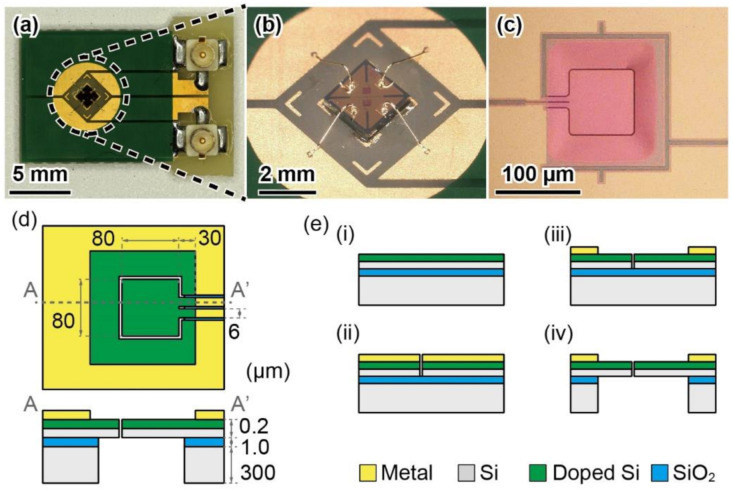
(**a**) Photograph of printed circuit board with sensor chip; (**b**) close-up view of sensor chip; (**c**) close-up view of piezoresistive cantilever. (**d**) Design of piezoresistive cantilever. (**e**) Fabrication process of piezoresistive cantilever: (**i**) Formation of piezoresistive layer on device Si layer; (**ii**) deposition and patterning of Au/Cr layer and etching of device Si layer; (**iii**) patterning of Au/Cr layer; (**iv**) etching of handle Si layer and SiO_2_ layer.

Figure 4a–c shows a photograph of a printed circuit board (PCB) with a sensor chip, a close-up view of the sensor chip, and a close-up view of a piezoresistive cantilever, respectively. The sensor chip measured 1.5 mm × 1.5 mm × 0.3 mm. The piezoresistive cantilever was placed at the center of the chip. The design of the cantilever is shown in Figure 4d. The cantilever measured 80 μm × 80 μm × 0.2 μm. The leg length was approximately 30 μm. The gap surrounding the cantilever was approximately 1 µm. To compensate for the temperature, a dummy cantilever was formed next to the pressure sensing cantilever.

The fabrication process of the MEMS piezoresistive cantilever is illustrated in Figure 4e. The detailed fabrication process has been described previously [28]. The piezoresistive cantilever was formed on a silicon-on-insulator wafer of which device Si layer, SiO_2_ layer, and handle Si layer were 0.2 μm, 1 μm, and 300 μm, respectively. First, an n-type piezoresistive layer was formed on the device Si layer, as shown in Figure 4e(i). Second, an Au/Cr layer was deposited and patterned on the device Si layer. Next, the device Si layer was etched via inductively coupled plasma reactive ion etching (Figure 4e(ii)). Subsequently, the Au/Cr layer was re-patterned (Figure 4e(iii)). Finally, the handle Si layer was etched and the SiO_2_ layer was removed (Figure 4e(iv)). For post-processing, the sensor chips were released from the wafer. The initial resistances of the sensing and dummy cantilevers were approximately 5 kΩ.

## 3. Experiment and Results

### 3.1. Differential Pressure Calibration

We calibrated the sensor chips by measuring the change in resistance in response to the differential pressure. The sensor chip was attached to a PCB at the position where the penetration hole was formed, as shown in Figure 4a. Subsequently, the sensor chip was wire bonded to the PCB for electrical connection. The two cantilevers were connected to a bridge circuit to measure the resistance change as a change in voltage via an instrumentation amplifier circuit. We set the gain of the instrumentation amplifier *A* and bridge voltage *V*_IN_ to 100 and 1 V, respectively. Hence, the relationship between the voltage change Δ*V* and fractional resistance change Δ*R/R* can be expressed as follows:

(19) $$\Delta V=A\cdot V_{\mathrm{IN}}\cdot \frac{\Delta R}{4R}=25\frac{\Delta R}{R}$$

**Figure 5:**
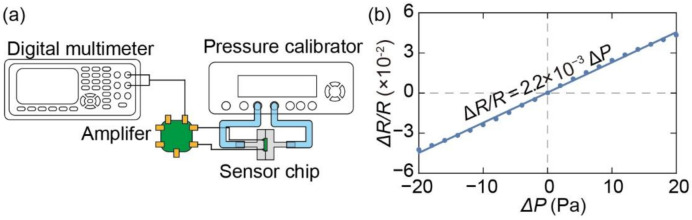
(**a**) Method of calibrating piezoresistive cantilever against differential pressure. (**b**) Relationship between differential pressure and fractional resistance change.

Figure 5a shows the experimental setup for the differential pressure calibration. The sensor substrate was sandwiched between two air chambers fabricated using a 3D printer (Formlabs, Form 3, Somerville, MA, USA) [29]. The chambers were connected to a pressure calibrator (Halstrup–Walcher GmbH, KAL 200, Kirchzarten, Germany) using silicone tubes. Static differential pressure was applied to the sensor from the pressure calibrator from −20 Pa to +20 Pa at intervals of 2 Pa. The output voltage was measured using a digital multimeter (KEYSIGHT, 34465A, Santa Rosa, CA, USA). The relationship between the applied voltage and fractional resistance change of the piezoresistive cantilever is shown in Figure 5b. The fractional resistance change responded linearly to the differential pressure. Sensitivity (∆*R/R)/*∆*P* was calculated as follows:

(20) $$\left(\frac{\Delta R}{R}\right)/\Delta P=2.2\times 10^{-3}{\;\mathrm{Pa}}^{-1}$$

Subsequently, the differential pressure resolution, which was defined from the noise level as ∆*R*_RMS_/*R*, was calculated to be approximately 0.033 Pa.

### 3.2. Air Leakage Test

**Figure 6:**
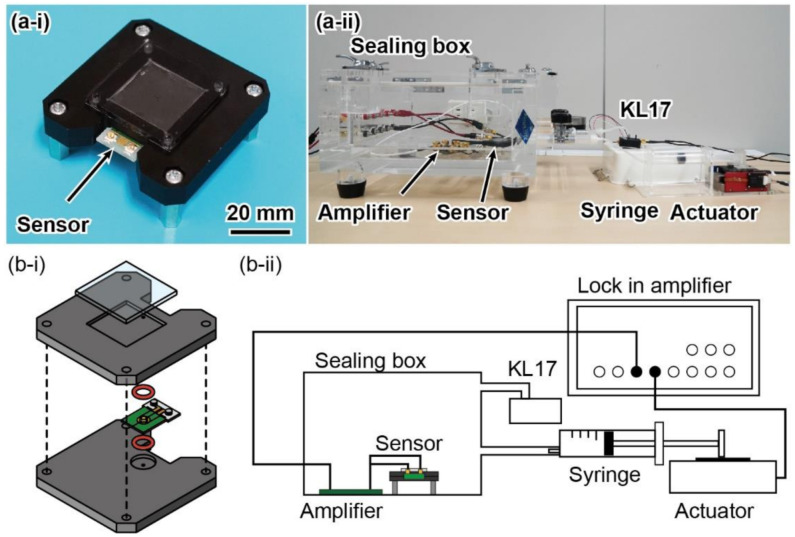
(**a**) Photographs and (**b**) illustration of (**i**) sensor component and (**ii**) whole calibration setup for evaluating air leakage of piezoresistive cantilever.

We experimentally evaluated the dynamic pressure characteristics of a piezoresistive cantilever to obtain the constant *k* of the air leakage. The experimental setup, which was based on a previous study, is shown in Figure 6 [30]. The sensor unit component for calibration was composed of a piezoresistive cantilever and an air chamber, as shown in Figure 3a. To develop such a sensor unit, the PCB sensor substrate was sandwiched between two metal plates with penetration holes, as shown in Figure 6(a-i,b-i). One plate was opened to the external atmosphere, whereas the other plate functioned as an air chamber with a volume *V*_3_ of 0.63 mL. The plate had square grooves, which allowed an air-sealed chamber to be formed by attaching a flat panel. The sensor unit was fixed inside a sealing box measuring 300 mm × 300 mm × 100 mm, which enabled a dynamic pressure change Δ*P*_atm_ to be applied, as shown in Figure 6(a-ii,b-ii). The experimental setup comprised a sealing box, syringe driven by a linear actuator, calibration pressure sensor (Nagano Keiki, KL17, Nagano, Japan), and lock-in amplifier (Zurich Instruments, MFLI, Zurich, Switzerland). The calibration pressure sensor was used to measure the pressure change Δ*P*_atm_. The syringe was connected to the sealing box via a silicone tube such that the pressure inside the box varied sinusoidally when the syringe was driven to propagate back and forth sinusoidally via the output signal of the lock-in-amplifier. Subsequently, the responses of the sensor unit and calibration sensor were measured using a lock-in-amplifier or an oscilloscope (Yokogawa Electric Corporation, DL350, Musashino, Tokyo, Japan). We used the same amplifier circuit for the differential pressure calibration.

**Figure 7:**
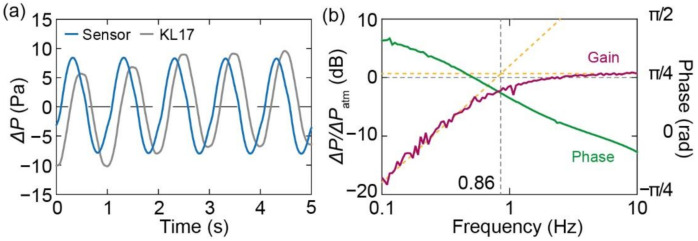
(**a**) Output waveform for applied frequency of 1 Hz. (**b**) Frequency response of sensor unit.

Figure 7a shows the response waveforms at 1 Hz recorded by the oscilloscope. It was confirmed that the responses of both sensors were sinusoidal. In the experiment, frequency responses were obtained using 100-point log sweeps over an input frequency range from 0.1 to 10 Hz. Pressure changes of approximately 15 Pa (peak-to-peak) were applied at all frequencies.

Figure 7b shows the Bode plot of the sensor units obtained based on the measurement results. The gain and phase were defined as the ratio of the pressures measured by the piezoresistive cantilever and calibration sensor, which is the same as that expressed in Equation (18). Both the gain and phase of the sensor show characteristics similar to the theoretical results shown in Figure 3b; the frequency characteristics reflect those of a high-pass filter, and the gain converged to zero. Therefore, the cutoff frequency of the high-pass filter was obtained as the intersection of the slope and convergence lines, as shown by the dotted orange lines in Figure 7b. The calculated frequency was approximately 0.86 Hz. Using the measured cutoff frequency (0.86 Hz), chamber volume (0.63 mL), and atmospheric pressure (0.1 MPa), the constant *k* due to air leakage was calculated to be 5.3 × 10^−12^ Pa^−1^·m^3^/s.

### 3.3. Pulse Wave Sensor Development

**Figure 8:**
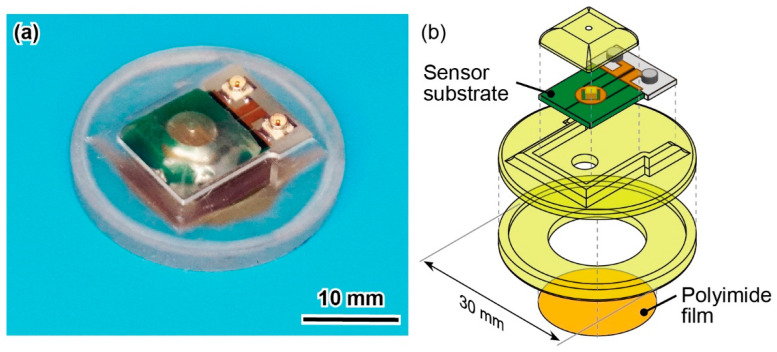
(**a**) Photograph and (**b**) schematic illustration of developed pulse wave sensor.

A pulse wave sensor was fabricated using a piezoresistive cantilever element, for which the constant *k* was determined. Based on Equation (12), the optimal sensor chamber volume *V*_2_ for the pulse wave measurement using the cantilever was calculated to be 0.77 mL. Using this value, we designed and developed a pulse wave sensor, as shown in Figure 8. The sensor was composed of a calibrated sensor substrate, jigs for air chambers, and a polyimide film, as shown in Figure 2a. The jigs were fabricated using a 3D printer, similar to the differential pressure calibration, and attached to the sensor substrate using an adhesive. The sensor measured 30 mm in diameter and 8 mm in thickness, including the upper chamber. The upper chamber had a hole with a diameter of 1 mm on the top surface, which enabled an infinite volume to be achieved. A 15-mm-diameter hole was created in the lower chamber to allow a 50-μm-thick polyimide film to be affixed such that the hole is covered to form the attachment portion to the skin.

**Figure 9:**
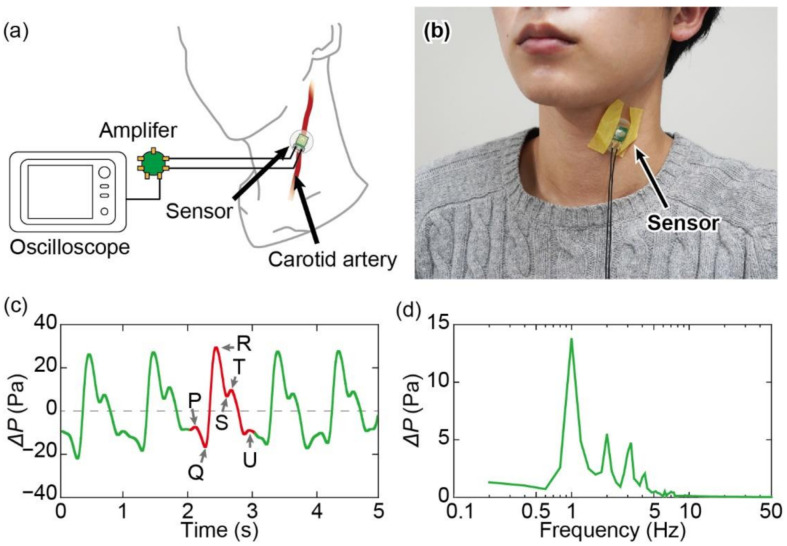
(**a**) Illustration and (**b**) photograph of measurement of pulse wave using developed sensor. (**c**) Response of sensor to pulse wave. (**d**) FFT analysis of measured signal.

As a demonstration, we measured the pulse wave at the carotid artery using the developed sensor, as shown in Figure 9a,b. The sensor was fixed onto the skin above the carotid artery using masking tape, and the sensor output was recorded using an oscilloscope via an amplifier circuit. The sensor was attached to the skin using only tape to avoid a high pressing force.

The pulse wave measured for 5 s, as shown in Figure 9c, was applied using a low-pass filter whose frequency was 30 Hz. The measurements were conducted while a 22-year-old healthy male participant was in a resting state, with a pulse rate of approximately 1 Hz. The measured pressure varied by ±20 Pa. The signal level was approximately 1000 times greater than the sensor resolution. It is noteworthy that the pressure change does not reflect a direct variation in the blood pressure but is a response to the chamber pressure change from skin movement. When focusing on a single cycle of the pulse wave signal, six characteristic peaks were observed from the P to U waves, and the time lag between those peaks is an important medical criterion [2,3]. The measured pulse waves reflected the peak waves in every cycle. Unlike the conventional pulse wave measured via electrocardiography, the S and T waves shifted upward in the graph as compared with the P, Q, and U waves.

Figure 9d shows the fast Fourier transform (FFT) analysis of the measured pulse wave. The signal was the largest at 1 Hz, which corresponds to the frequency of a heartbeat. In addition, peaks appeared in the second-and third-harmonic frequencies. By contrast, the low-frequency components of the measured pulse wave were approximately 0.8 Hz. This indicates that the sensor exhibits suitable frequency characteristics for pulse-wave measurements.

## 4. Discussion

Although the frequency characteristics of the sensor were designed based on the constant air leakage of the cantilever and the chamber volume, the actual characteristics of the fabricated device were not evaluated directly. It is difficult to measure the sensor response with a constant volume change, defined as the pulse wave signal, while sweeping the frequency. Additionally, a polyimide film was used to convert the skin deformation caused by pulse waves into chamber volume changes; however, the response might vary depending on the softness of the film and target skin. If the film is stiff, then skin deformation is inhibited, and the signal is attenuated. In addition, the pressure change in the chamber was determined by the fractional volume change, as expressed in Equation (9). For a constant volume, the pressure change increases if the bottom area is larger and the height is smaller. In this study, we focused on the frequency response, not on the specific dimensions of the chamber or the mechanical properties of the film. In subsequent studies, these parameters should be evaluated to optimize the sensor.

Furthermore, the air leakage rate can be modified by changing the dimensions of the cantilever, although this results in a tradeoff with the sensitivity. Provided that the cantilever is downsized and the constant of air leakage is halved, the chamber size can be halved to obtain the same frequency characteristics. A smaller size is desirable for wearing the sensor for long periods without discomfort. Additionally, the sensor signal was measured while it was wired through an amplifier; however, by adopting wireless technology, it can function as a wearable sensor. It is relatively easy to realize a wireless system using the proposed sensor because the latter outputs a simple analog signal and does not consume a significant amount of power.

## 5. Conclusions

We realized a pulse wave sensor with appropriate frequency characteristics using a MEMS piezoresistive cantilever element and air chambers. By establishing contact between the chamber of the sensor with the skin via a soft film, the volume of the chamber changed owing to the film deformation caused by pulse waves. The pressure inside the chamber changed, and the pulse waves can be measured by detecting the pressure variation using a piezoresistive cantilever. The proposed sensor structure allows the initial pressure to be physically excluded by a high-pass filter when attaching the sensor to the skin owing to air leakage around the cantilever.

To enable a quantitative design of the sensor characteristics, we derived a theoretical equation for the sensor frequency response. It was discovered that the sensitivity and cutoff frequency of the high-pass filter depended on the air leakage ratio of the cantilever element and the air chamber volume. Hence, the required specification was set such that the cutoff frequency was 0.7 Hz to include the smallest frequency band of the pulse wave with the highest sensitivity.

To design the pulse wave sensor based on the theoretical equation, we first evaluated the piezoresistive cantilever for its differential pressure sensitivity and air leakage rate. The calibration results showed that the sensitivity and air leakage constant were Δ*R*/*R*/Δ*P* = 2.2 × 10^−3^ Pa and 5.3 × 10^−12^ Pa^−1^·m^3^/s, respectively. Next, we fabricated a prototype sensor using 3D printer jigs and a polyimide film based on the calibration results. The chamber volume was 0.77 mL, and the sensor measured 30 mm in diameter and 8 mm in thickness, which is sufficiently compact to be a wearable device.

Finally, as a demonstration, we measured the pulse waves at the carotid artery of a participant using the developed sensor. The measured pressure varied by ±20 Pa, which was approximately 1000 times greater than the sensor resolution. Focusing on the pulse characteristics, we confirmed that the important characteristics of the pulse waves were identifiable from the measured signal. Therefore, we believe that the results presented herein will facilitate the design of pulse wave sensors based on cantilever-type pressure sensors.

## Data Availability

The data presented herein are available upon request from the corresponding author.
